# The Character of a Veterinary Surgeon

**Published:** 1818

**Authors:** James Carver


### THE CHARACTER

OF A

### VETERINARY SURGEON.

THE character of a Veterinary Surgeon, in a limited sense, is one who practises the operative part of the Veterinary Art, and whose views do not extend to the treatment of constitutional maladies in brute animals.

The veterinary practitioners in general are named Veterinary Surgeons—and this designation also attaches to those who engage in all the branches of the profession, as they are required in the different regiments of cavalry. We shall devote this article particularly to the consideration of those qualifications which every man engaged in it ought to possess, in an equal degree with those whose conduct and operation are exercised on the human body.

There is undoubtedly no profession in which greater natural qualifications are required than our own. The more liberal nature has been in her gifts, the more carefully the first impressions have been cultivated by rational education—by so much the better will a man be fitted for the practice of it. *Youth, firmness, dexterity, acute sensation, sound judgment,* and *humanity,* are the qualifications which may be considered as necessary for a surgeon, whether his patient be a *man* or a *quadruped*.

We will begin by observing—that in

### YOUTH, strong impressions are made on the mind, and that he who begins to study on the brute as well as the human subject, from the earliest period of life, will be most likely to acquire reputation.

### FIRMNESS, is the second qualification of a Veterinary Surgeon, and is indeed extended to the mind as well as the body. It implies resolution to go through his operations, however hazardous or severe, undisturbed by any accidental circumstances, unmoved or unawed by the presence of spectators. It also implies presence of mind to determine how to act under all circumstances.

### DEXTERITY, in using his instruments, is also a necessary qualification in a Veterinary Surgeon. It enables him to finish an operation with all convenient despatch, and with the least pain to the patient, whether brute or human.

### ACUTE SENSATION, is extremely necessary also for a Veterinary Surgeon; for how often do instances occur in the acute diseases of the horse, where the nicest delicacy of the touch is necessary to distinguish the true state of the pulse.

### SOUND JUDGMENT, is, on many accounts, of the utmost importance to the Veterinarian. It enables him to form judicious prognostics, by which he may calculate the chances for or against the event of any operation proposed. It is often not less useful in deciding for the patient's possible advantage, than in preserving his own reputation, and keeping up the credit of his art. It also teaches him to determine with precision the time necessary for performing an operation, leads him to the choice of the best methods of executing it, or perhaps furnishes him with the *more laudable* and *happy contrivance* of recovery of his patient by more gentle means.

### HUMANITY, is the last qualification mentioned as necessary for a Veterinary Surgeon; and though last, not the least important and laudable.

This indeed is the cardinal qualification of all; it reflects a lustre on the rest, and completes the true character of the man as well as of the surgeon. The exercise of it is required in two ways; first, humanity in operation, and secondly, tenderness in our subsequent treatment. Humanity in operating, should induce us to put an end to our patient's sufferings, (whether brute or human) as soon as we can, and also to perform this severe though necessary task after such a manner as shall be attended with the least possible degree of pain, besides the pleasing satisfaction resulting to ourselves, of having done our duty when actuated by such motives.

Tenderness in our behaviour to the brute creation, needs not an argument to enforce its necessity—it being no less honourable to feel for them than ourselves; and surely the distresses of brute creatures, and the pain we are often obliged to inflict upon them, is sufficient to soften the hardest heart, and to raise the emotions of compassion within us towards those *mute sufferers* who have toiled in our fields, and lent the labouring hand to help build our cities and our churches.

When dressings are either removed or applied, it should be done with a gentle hand, and in a manner which would convince the bystander that it is not the veterinary surgeon's intention to give pain, even to the most inferior animal, if he can avoid it; while a contrary conduct to this may ever prove an obstacle to his success in life; for cruelty will increase by habit, and at length render his manners coarse and offensive, even to those on whose liberality the emoluments of his future practice may in a great measure depend.

We shall now come to consider the *acquired knowledge* necessary to make a good veterinary surgeon. On this point we shall make one general observation, to wit: that the more extensive and universal a man's knowledge may be, from having made these his pursuits and acquirements in various quarters of the globe, (and which the writer has had every opportunity of obtaining from early life) the better fitted will he be for the exercise of his profession. But, not to alarm young persons by considering the subject too extensively, or by a vain display of science, it is necessary here to mention that knowledge which it is absolutely necessary they should acquire. If they are as conversant as they ought to be, in the matter proposed to their industry and application in this work, the knowledge they will then have obtained cannot but raise a spirit of inquiry in their minds, which will lead to more important exertions.

The next and most important acquisition is a knowledge of the *power and properties of Medicines*. The various substances of the materia medica—the different classes of the *vegetable, mineral,* and *animal* kingdoms, so far as they relate to physic, supply all the several applications used in *Veterinary Surgery*. If, therefore, we are ignorant of the qualities of these substances, we may commit the greatest mistakes in the use of them. Instead of an emollient we may apply an escharotic, and instead of a stimulating application, we may perhaps prescribe a sedative.

Without this knowledge, it is impossible to practise our profession with any degree of credit or success; though by some it may possibly be argued that we should have learned these things equally from experience. Nothing therefore can be more necessary than a knowledge of the *Materia Medica*, and consequently of *Veterinary Pharmacy*—which is nothing more than a knowledge of the art of mixing and compounding the several articles of the *Veterinary Materia Medica*, so as to produce a combination capable of effecting what cannot be done by any solid or fluid substance *singly*.

The last point to be insisted on, as demanding our particular attention, is the study of anatomy. The body of the *Horse*, the *Cow*, the *Sheep*, and the *Dog*, being the subject of our operations, how shall we be able to perform them properly, if we are ignorant of the construction of the machine on which we are to work. *A complete and thorough knowledge of Comparative Anatomy*, it is therefore absolutely necessary to acquire; and the method to be pursued, in order to acquire this knowledge, must be the work of our own hands in the *dissecting rooms* of those institutions established for that purpose, in different parts of Europe. Mere oral instruction is not sufficient. We may attend the most ingenious and instructive lectures in anatomy of the *human subject*, without being fitted for the exercise of our profession. It is therefore necessary to dissect, to trace, and inspect, the several parts of animals with our own *hands* and *eyes;* and this with care and industry.

